# Enhancing the Physicochemical Properties, Bioactivity, and Functional Applications of Fresh Jujube Juice Using Media Milling

**DOI:** 10.1021/acsomega.5c00475

**Published:** 2025-03-18

**Authors:** Hong-Yi Kang, An-I Yeh, Min-Hsiung Pan

**Affiliations:** † Institute of Food Science and Technology, National Taiwan University, Taipei 10617, Taiwan; ‡ Department of Medical Research, China Medical University Hospital, China Medical University, Taichung 40402, Taiwan

## Abstract

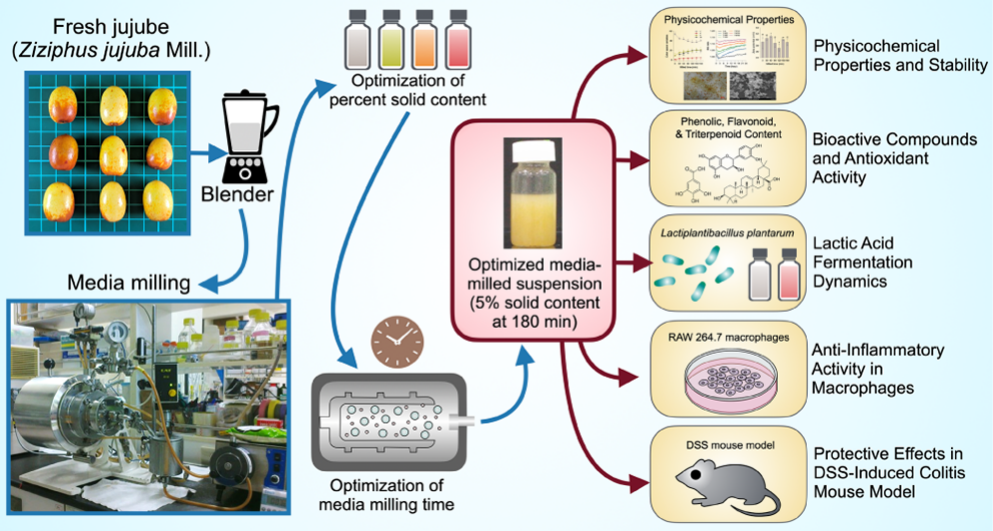

This study systematically evaluated the effects of media milling on the physicochemical properties, bioactive compound content, and functional applications of fresh jujube (*Ziziphus jujuba* Mill.) juice. Optimization experiments identified ideal conditions for nanoparticle production, including 5% solid content and a 180 min milling duration, resulting in significantly reduced particle sizes—volume-weighted average diameter (from 229.0 ± 1.0 to 25.0 ± 0.2 μm) and number-weighted average diameter (from 7.2 ± 0.0 to 0.1 ± 0.0 μm)—and improved dispersion stability. Media milling enhanced key physicochemical properties such as zeta potential, viscosity, and suspension stability, while also modifying color and pH. The process notably increased the content of bioactive compounds, including total flavonoids (from 2.9 ± 0.1 to 3.8 ± 0.0 mg catechin equivalent (CE)/g dry weight (DW)) and triterpenoids (from 15.4 ± 1.2 to 28.0 ± 4.9 mg oleanolic acid equivalent (OAE)/g DW). The antioxidant activity before and after media milling, assessed using 2,2-diphenyl-1-picrylhydrazyl (DPPH), ferric reducing antioxidant power (FRAP), and 2,2’-azino-bis(3-ethylbenzothiazoline-6-sulfonic acid) (ABTS) assays, remained comparable. Fermentation with *Lactiplantibacillus plantarum* demonstrated that both blended and media-milled jujube juice can serve as effective substrates for substrate utilization and lactic acid production. Anti-inflammatory assays using RAW 264.7 macrophages revealed reduced nitric oxide production and lower levels of pro-inflammatory cytokines such as IL-1β, showcasing the juice’s potential to modulate inflammation. In a dextran sodium sulfate (DSS)-induced colitis mouse model, media-milled jujube juice demonstrated safety, though it did not show significant protective effects. These findings position media-milled jujube juice as a promising functional food ingredient with potential applications in health promotion and disease management.

## 1 Introduction

Jujubes (*Ziziphus jujuba* Mill.), also known as Da Zao, Chinese dates, or red dates (when dried), are perennial deciduous fruit trees belonging to the Rhamnaceae family.^1^ Their composition and quality are significantly influenced by factors such as soil conditions, climate, cultivation methods, storage, and processing techniques.^2,3^ Fresh jujubes are rich in bioactive compounds, including polyphenols, flavonoids, triterpenes, polysaccharides, and cyclic adenosine monophosphate (cAMP), which exhibit antioxidant, anti-inflammatory, immune-boosting, and hepatoprotective properties.^4−6^ Studies in animal models have further demonstrated their potential to modulate gut microbiota, alleviate inflammation, and improve gastrointestinal health.^7,8^ Additionally, jujubes have higher levels of dietary fiber, sugar, protein, and pectin compared to many other fruits, enhancing their versatility in processing.^9,10^

Despite these benefits, preserving fresh jujubes has historically posed challenges, limiting their applications and often relegating them to dried forms commonly used in Traditional Chinese Medicine (TCM).^11^ In TCM, jujube fruits are used to tonify the spleen and stomach, nourish the blood, and calm the mind. They are traditionally used to treat fatigue, digestive disorders, and insomnia, while modern research confirms their antioxidant, anti-inflammatory, and immunomodulatory properties.^1^ The growing demand for functional foods and nutraceuticals has highlighted the potential of jujubes due to their nutritional and medicinal properties. However, the perishability of fresh jujubes and the limitations of traditional processing methods, such as sun-drying, often result in nutrient loss, reduced bioactivity, and limited product diversity, which affect the overall market value of jujube-derived products.^12,13^ Addressing these challenges necessitates the development of advanced processing technologies that retain nutritional integrity while expanding product applications.

Wet stirred media milling (WSMM) is a novel processing technique that employs mechanical force to reduce particle size and enhance the physicochemical properties of materials. Through shear, friction, and impact forces, WSMM increases particle surface area, improves water absorption, and facilitates the release of water-insoluble components.^14,15^ This technique has been widely applied in the food and pharmaceutical industries, including the production of stable suspensions and functional foods.^16−19^ Applications include innovations in soy milk processing, Ganoderma-based products, and high-performance chitin films.^19−21^ Compared to traditional milling methods, WSMM offers superior efficiency in enhancing the bioavailability of nutrients and functional compounds, making it particularly suitable for bioactive-rich fruits like jujubes.

Jujubes, like other fruits, are also excellent substrates for lactic acid bacteria (LAB) fermentation due to their high carbohydrate, dietary fiber, vitamin, and antioxidant content.^22^ LAB fermentation enhances the flavor, texture, and shelf life of products while releasing bound polyphenols and flavonoids, thereby improving their antioxidant activity. Jujube polysaccharides, in particular, provide an ideal growth substrate for probiotics, further enhancing the health benefits of fermented products.^23−25^ Recent studies have demonstrated that combining fermentation with pretreatment methods like milling can significantly improve bacterial growth and the overall quality of fermented products.^16^ However, limited research has explored the synergistic effects of WSMM and LAB fermentation on jujube-based products, representing a critical gap in the literature.

This study aims to prepare a jujube suspension using WSMM, investigate its physicochemical properties, bioactive compound content (polyphenols, flavonoids, and triterpenes), and antioxidant capacity, and explore its potential for LAB fermentation. By addressing the challenges of jujube preservation and product diversification, this research aligns with global efforts to develop sustainable food systems, reduce waste in fruit processing, and create high-value functional foods. The findings will provide valuable insights into the application of WSMM in fruit processing and fermentation, contributing to the enhancement of jujube product quality and market potential.

## 2 Materials and Methods

### 2.1 Materials

Chinese jujubes (*Ziziphus jujubaMill. Var inermis* (Bge.) Rehd.) were harvested during July–August from Longxi Farm, Gongguan Township, Miaoli County, Taiwan, under temperate climatic conditions with well-drained soil. These conditions are known to influence fruit composition, particularly the bioactive compound content. The fruits were stored at −20 °C and underwent thawing, washing, and chopping before use.

### 2.2 Preparation of Media-Milled Jujube Suspension

Fresh jujube fruits were deseeded, weighed, and combined with deionized water. To prepare the coarse jujube suspension, the fruits were ground using a Blender 7012S (Waring Commercial, USA) equipped with a cooling system and agitated for 5 min to form a slurry. The slurry was transferred to a 500 mL beaker, and the remaining material was rinsed with deionized water, designated as fresh fruit coarsely chopped material (F–B).

For media-milled suspensions, the milling process involved using a media mill (MiniPur, Netzsch-Feinmahltechnik GmbH, Selb, Bavaria, Germany) with 0.8 mm yttria-stabilized tetragonal zirconia (YTZ) beads loaded at 70% (v/v) in a 200 mL milling chamber.^26^ Fresh jujube and deionized water were milled at a rotational speed of 3000 rpm for a duration of 180 min under a 0.94 kW driving motor.

### 2.3 Physicochemical Characterization

#### 2.3.1 Particle Size Analysis

Particle size was measured using a static laser light diffraction particle size analyzer (LS230, Beckman Coulter Inc., Brea, CA, USA) with a detection range of 0.04–2000 μm. The volume-weighted (D[4,3]) and number-weighted (D[1,0]) particle diameters were calculated. Measurements were performed in triplicate. The average particle diameters were determined using the following equations:

Volume-weighted diameter (D[4,3]):
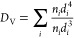


Number-weighted diameter (D[1,0]):
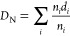


Where n_i_ is the number of particles of diameter d_i_.

#### 2.3.2 Morphological Observation

For optical microscope observation, Jujube peel samples were cut into appropriate sizes, and the jujube suspension was diluted 10-fold with deionized water. A drop of the diluted sample was placed on a glass slide using a pipet, and a coverslip was applied at a 45° angle to minimize air bubbles. Excess liquid was removed with lens paper. Observations were made under an optical microscope (Optiphot-Pol, Nikon Co., Tokyo, Japan). For scanning electron microscope (SEM) observation, Jujube suspension was diluted approximately 100-fold with deionized water. Samples were sequentially dehydrated using ethanol solutions at concentrations of 50%, 60%, 70%, 80%, 90%, and 100%, followed by a final dehydration step with 100% acetone. Critical point drying (CPD) was performed on the samples, which were then mounted onto aluminum stubs with double-sided conductive carbon tape. The dried samples were sputter-coated with gold using an ion sputtering device (E-101, Hitachi Co. Ltd., Japan) under vacuum. Morphological observations were conducted using a scanning electron microscope (SEM, Hitachi S-4800, Hitachi Co. Ltd., Japan) at an acceleration voltage of 15 kV and a magnification of 1,000×.

#### 2.3.3 Zeta Potential (ζ-Potential)

Samples were diluted in deionized water and analyzed using a Zetasizer ZS90 (Malvern Instrument Inc., Worcestershire, UK) to measure particle charge potential.

### 2.4 Suspension Stability

Stability was evaluated using a demixing tester (LUMiCheck, L.U.M. GmbH, Berlin, Germany). Backscattered light intensity at 870 nm was recorded over 24 h. Stability was quantified by calculating the backscattering ratio (BS ratio) and percentage aggregation (%At):

BS ratio:
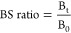


Percentage aggregation (At):
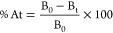


Here, B_t_ represents the light intensity at time t_i_, and B_0_ represents the initial light intensity at 0 min.^27^

### 2.5 Color Analysis

Color space variables (L*, a*, b*) were measured using a Color Meter ZE 2000 (NIPPON DENSHOKU INDUSTRIES CO., Ltd., Tokyo, Japan). Chroma values were calculated as
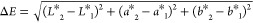


### 2.6 Viscosity

Viscosity was measured at a shear rate of 100 s^–1^ using a dynamic rheometer (AR-2000ex, TA Instruments, Inc., New Castle, DE, USA).

### 2.7 Bioactive Compound Analysis

For total phenolic content (TPC), total flavonoid content (TFC) and total triterpenoid content (TTC) analysis, both juice samples were centrifuged at 10000 × *g* for 10 min, and the pellets were discarded. At the same time, the supernatants were filtered with 5 μm pores filter, followed by freeze-drying. 100 mg of freeze-dried jujube powder was dissolved in 1 mL of deionized water, thoroughly mixed and stored as stock.

#### 2.7.1 Total Phenolic Content (TPC)

TPC was determined using the Folin–Ciocalteu method and expressed as mg gallic acid equivalent (GAE)/g dry weight (DW).^28^

#### 2.7.2 Total Flavonoid Content (TFC)

TFC was measured using the aluminum chloride colorimetric method and expressed as mg catechin equivalent (CE)/g DW.^29^

#### 2.7.3 Total Triterpenoid Content (TTC)

TTC was assessed using the vanillin-perchloric acid assay and expressed as mg oleanolic acid equivalent (OAE)/g DW.^30^

### 2.8 High-Performance Liquid Chromatography (HPLC) Analysis for Triterpenoid Quantification

HPLC Conditions: HPLC analysis was conducted using a Waters SunFire C18 column (250 mm × 4.6 mm, 5 μm) with a UV–vis detector set at 210 nm. The mobile phase consisted of solvent A (methanol) and solvent B (water with 0.08% phosphoric acid, pH 3). The gradient elution program was as follows: initially, 55% solvent A and 45% solvent B were maintained for the first 16 min. The proportion of solvent A was then increased to 65% at 36 min, 77% at 46 min, and 80% at 56 min. The gradient reached 95% solvent A at 70 min and was maintained until 90 min. Finally, the gradient returned to the initial condition of 55% solvent A and 45% solvent B at 95 min and was held until 100 min. The flow rate was set at 0.6 mL/min, and the column temperature was maintained at 25 °C. The injection volume for all samples was 10 μL, and the total run time was 100 min.

Preparation of Standard Curve: A stock solution of triterpenoid standards (betulinic acid, oleanolic acid, and ursolic acid) (200 μg/mL) was prepared in methanol. Serial dilutions were made to obtain working concentrations of 100, 50, 25, 12.5, and 6.25 μg/mL. Approximately 2 mL of each diluted solution was filtered through a 0.45 μm nylon membrane filter and transferred to amber sample vials. HPLC injections were performed using an autosampler with an injection volume of 10 μL. Peak areas (mV·min) were recorded and plotted against triterpenoid concentrations to generate a standard curve. The coefficient of determination (R^2^) was required to be greater than 0.99 to ensure accurate quantification.^31^

### 2.9 Antioxidant Activity

Antioxidant capacity was determined using DPPH radical scavenging activity,^32^ reducing power,^33^ Trolox equivalent antioxidant capacity (TEAC),^34^ and ferric reducing antioxidant power (FRAP).^35^

### 2.10 Cell Culture and Viability Assay (RAW 264.7 Cell Line)

RAW 264.7 cells, derived from BALB/c mouse monocytes (ATCC NO. TIB-71), were purchased from the Bioresource Collection and Research Center (BCRC, Taiwan). RAW 264.7 cells were cultured in Dulbecco’s Modified Eagle Medium (DMEM) supplemented with 10% fetal bovine serum and 100 U/mL penicillin-streptomycin.^36,37^ Jujube suspension supernatant was freeze-dried to a powder form, reconstituted with dimethyl sulfoxide (DMSO) to the desired concentration, and filtered through a 0.22 μm membrane filter under sterile conditions. RAW 264.7 cells were seeded at a density of 1 × 10^5^ cells/well in 96-well plates and allowed to adhere overnight. Cells were treated with 250, 500, and 1000 μg/mL of the samples for 24 h.

Cell viability was assessed using the MTT assay. Briefly, after 24 h of treatment, 10 μL of MTT solution (5 mg/mL in PBS) was added to each well, and the plates were incubated at 37 °C for 4 h. The formazan crystals formed were dissolved in 100 μL of DMSO, and the absorbance was measured at 570 nm using a microplate reader (BioTek Instruments, Winooski, VT, USA).^38^

Nitric oxide (NO) production was measured using the Griess reagent assay. After 24 h of treatment, 50 μL of culture supernatant was mixed with an equal volume of Griess reagent (1% sulfanilamide and 0.1% *N*-(1-naphthyl) ethylenediamine dihydrochloride in 2.5% phosphoric acid). The mixture was incubated at room temperature for 10 min, and the absorbance was measured at 540 nm. NO concentrations were determined by comparing the absorbance values to a standard curve constructed using sodium nitrite.^39^

Cytokine levels (TNF-α, IL-1β, and IL-6) were quantified using enzyme-linked immunosorbent assay (ELISA) kits (BD Biosciences, San Jose, CA, USA) following the manufacturer’s instructions. Each sample was analyzed in triplicate, and absorbance was read at 450 nm with a reference wavelength of 570 nm. The cytokine concentrations were calculated based on standard curves generated from serial dilutions of recombinant standards provided with the kits.^40^

### 2.11 Lactic Acid Bacteria Fermentation

The lactic acid bacteria (LAB) strain utilized in this study was *Lactiplantibacillus plantarum* subsp. *plantarum* (BCRC 10069, Taiwan; ATCC 14917, USA), which was cultured at 37 °C in Lactobacilli MRS Broth (DIFCO 0881) under aerobic conditions. The strain was first activated by culturing in MRS broth for 24 h to reach the logarithmic growth phase before use in fermentation experiments.

Fresh jujube suspension was pasteurized at 80 °C for 15 min to eliminate contaminants while preserving bioactive compounds. After cooling to 37 °C, 10% (v/v) of the activated *Lactiplantibacillus plantarum* culture was inoculated into the jujube suspension. The fermentation process was carried out at 37 °C for 48 h under static conditions.

Samples were collected at 0, 12, 24, 36, and 48 h for analysis. Soluble solids (°Brix) were measured using a hand-held refractometer (Atago Co., Tokyo, Japan), while glucose concentrations were determined using the ASK Glucose Liquid Reagent (Tonyar Biotech, Inc., Taiwan). The pH was monitored using a pH meter (SP-2200, Suntex Instruments, Taipei, Taiwan), and titratable acidity was expressed as a percentage of lactic acid equivalent.

The consumption of soluble solids and reducing sugars by LAB was monitored throughout the fermentation process to assess the metabolic activity of *Lactiplantibacillus plantarum* and the efficiency of substrate utilization.

### 2.12 DSS-Induced Colitis Animal Model

Five-week-old male BALB/c mice (16–18 g) were obtained from the National Laboratory Animal Center (Taipei, Taiwan) and housed in the Experimental Animal Resource Center at National Taiwan University at 25 ± 1 °C with 50% relative humidity under a 12-h light/dark cycle (IACUC Approval No: NTU-107-EL-00222). Mice had free access to standard rodent chow (Laboratory Rodent Diet 5001) and autoclaved water.

Mice were acclimated for 1 week and randomly divided into five groups (*n* = 10 per group): (1) Control group (CON)—received no treatment; (2) DSS group (DSS)—received 2% DSS (dextran sulfate sodium) in drinking water from day 18 to day 24; (3) DSS + F–B group—gavaged daily with 0.1 mL of coarsely ground fresh jujube suspension; (4) DSS + F-M group—gavaged daily with 0.1 mL of media-milled fresh jujube suspension; and (5) DSS + F–S group—gavaged daily with 0.1 mL of the supernatant from the media-milled fresh jujube suspension.

Jujube suspensions were freshly prepared each week and administered by oral gavage once daily until day 27. From day 18 to day 24, colitis was induced in the DSS groups by administering 2% DSS (MW: 36,000–50 000 Da; MP Biomedicals, USA) in drinking water for seven consecutive days, followed by regular drinking water until the end of the experiment on day 28.

Body weight, stool consistency, and rectal bleeding were monitored daily. Mice were sacrificed on day 28 by CO_2_ asphyxiation, and blood was collected via cardiac puncture for further analysis. Colon tissues were excised, and their lengths were measured from the cecum to the rectum.

### 2.13 Statistical Analysis

All statistical analyses were carried out using GraphPad Prism (version 10.4.0). Data were expressed as the mean ± standard deviation of triplicate measurements. Statistical differences were determined using one-way ANOVA or two-way repeated measures ANOVA followed by Tukey’s multiple comparisons tests (*p* < 0.05).

## 3 Results

### 3.1 Optimization of Nanoparticle Preparation in Fresh Jujube Juice

**Figure 1:**
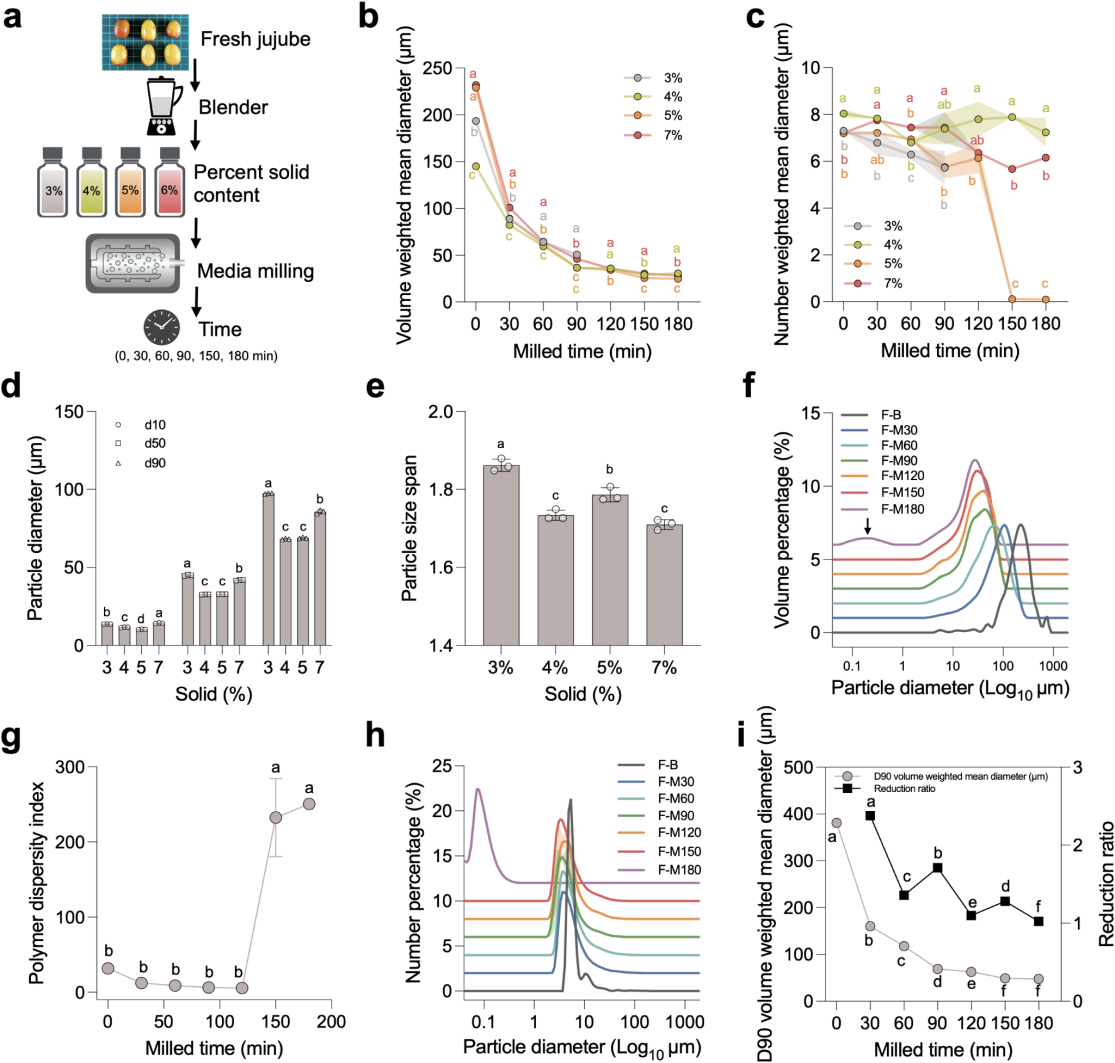
Optimization of nanoparticle preparation in fresh jujube juice using blending and media milling. Fresh jujube juice nanoparticles were prepared using a combination of blending and media milling, with a solid content of 5% in blended fresh jujube identified as the optimal concentration for achieving the smallest particle size and selected for further investigation, while a milling duration of 180 min was determined to be ideal for nanoparticle production. The figure illustrates (a) the experimental design and the effects of fresh jujube juice solid concentration and milling time on (b) volume-weighted average diameter, (c) number-weighted average diameter, (d) particle size distribution, (e) particle size span, (f) volume percentage, (g) polymer dispersity index (PDI), (h) number percentage, and (i) D90 volume-weighted mean diameter and reduction ratio. Statistical analyses were performed using one-way ANOVA with Tukey’s multiple comparisons tests, comparing different groups at each time point for (b) and (c), between groups for (d) and (e), and across time points for (g) and (h). Different letters indicate significant differences (*p* < 0.05) among groups. Data are presented as mean ± SD.

Fresh jujube juice nanoparticles were optimized using a combination of blending and media milling, focusing on solid content and milling duration (Figure 1a). A solid content of 5% was identified as optimal, yielding the smallest particle size. Volume-weighted mean diameter (D[4,3]) and number-weighted mean diameter (D[1,0]) progressively decreased with milling time, with significant reductions observed up to 180 min (Figure 1b,c). The volume-weighted average diameter was 229.0 ± 1.0 μm and decreased to 25.0 ± 0.2 μm, while the number-weighted average diameter changed from 7.2 ± 0.0 μm to 0.1 ± 0.0 μm. This indicates that prolonged milling effectively breaks down larger particles into more uniform nanoparticles. Particle size distribution (d10, d50, d90) was narrowest at 5% solid content (Figure 1d), and particle size span decreased (Figure 1e), reflecting improved uniformity and better dispersion. Volume percentage and number percentage analyses further supported enhanced dispersion, with the reduction ratio of D90 stabilizing after 150 min (Figure 1f–i). Additionally, the presence of nanoparticles was observed at the 180 min milling duration. These findings established the optimal milling parameters for nanoparticle production, with a 5% solid content and 180 min milling duration selected for subsequent experiments.

### 3.2 Physicochemical Properties and Stability of Media-Milled Jujube Juice

**Figure 2:**
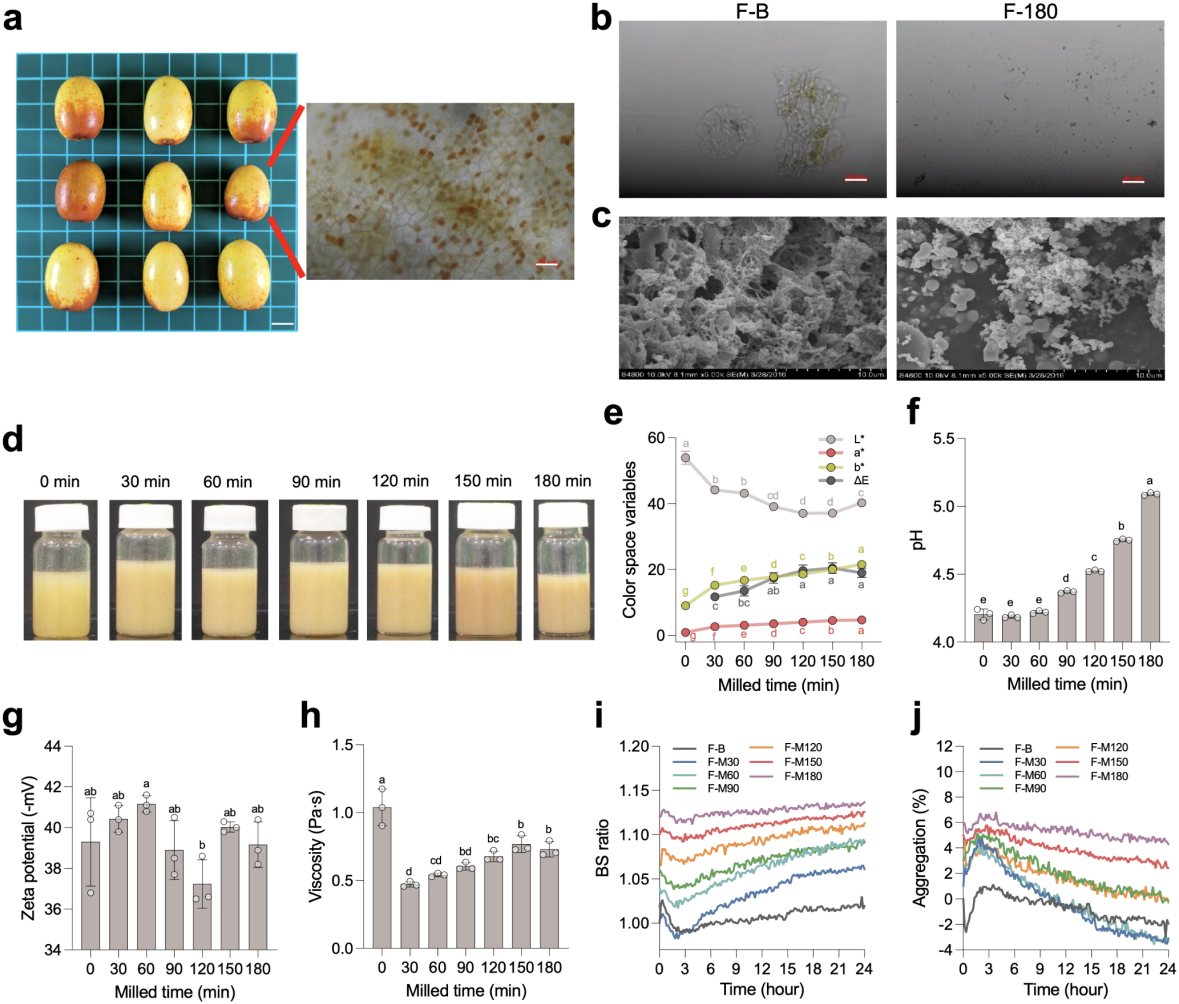
Effect of media milling on fresh jujube juice nanoparticles: appearance, microstructure, color, physicochemical properties, and stability. (a) The visual appearance of fresh jujube fruits (scale bar: 1 cm) and a microscopic image showing the cellular structure (scale bar: 50 μm), (b) optical microscopy images of jujube juice before and after media milling, illustrating particle size reduction, (c) scanning electron microscopy (SEM) images highlighting the changes in microstructure after media milling, (d) photographs of jujube juice at different milling times (0–180 min), showing color and dispersion changes, (e) color space variables (L*, a*, b*, and ΔE) of jujube juice samples as a function of milling time, indicating color changes with increased milling duration, (f) pH values of jujube juice samples at various milling times, showing a gradual increase over time, (g) zeta potential measurements demonstrating the effect of milling time on the surface charge of nanoparticles, (h) viscosity of jujube juice as a function of milling time, showing an increase with longer milling durations, (i) backscattering (BS) ratio profiles during 24-h storage, comparing stability across milling times, (j) aggregation percentage of jujube juice samples during 24-h storage, indicating improved stability with increased milling time. Statistical analyses were performed using one-way ANOVA with Tukey’s multiple comparisons tests, comparing the changes across time points for (e), (f), (g), and (h). Abbreviations: Blended fresh jujube (F–B); media-milled fresh jujube for 180 min (F-M180). Different letters indicate significant differences (*p* < 0.05) among groups. Data are presented as mean ± SD.

The physicochemical properties of media-milled jujube juice were assessed to evaluate the effects of nanoparticle production (Figure 2). Optical microscopy images (Figure 2b) show a clear reduction in particle size in media-milled jujube juice compared to blended juice, with media-milled samples exhibiting a more uniform and dispersed particle distribution. Scanning electron microscopy (SEM) images (Figure 2c) further confirm these observations, revealing that media-milled juice has a smoother surface morphology and fewer aggregated structures, while the blended juice retains larger, irregular, and clustered particles. These morphological changes indicate that media milling effectively reduces particle size and improves the homogeneity of the suspension. Visual inspection of jujube juice at different milling times showed improved dispersion and a more uniform suspension with longer milling durations (Figure 2d). Color space variables (L*, a*, b*, ΔE) demonstrated noticeable changes in color with increased milling time, likely due to the exposure of intracellular components during milling (Figure 2e). The pH of the media-milled juice increased gradually with milling time, potentially reflecting the release of buffering components from jujube particles (Figure 2f). Zeta potential measurements indicated improved nanoparticle surface charge stability, which contributes to the enhanced suspension stability observed in the media-milled samples (Figure 2g). Viscosity increased with milling time, correlating with enhanced dispersion and the breakdown of insoluble components (Figure 2h). Stability analysis using backscattering (BS) ratios and aggregation percentages confirmed improved suspension stability with longer milling durations, particularly at 180 min, ensuring that media-milled juice remains homogeneous over time (Figure 2i,j).

### 3.3 Bioactive Compounds and Antioxidant Activity

**Figure 3:**
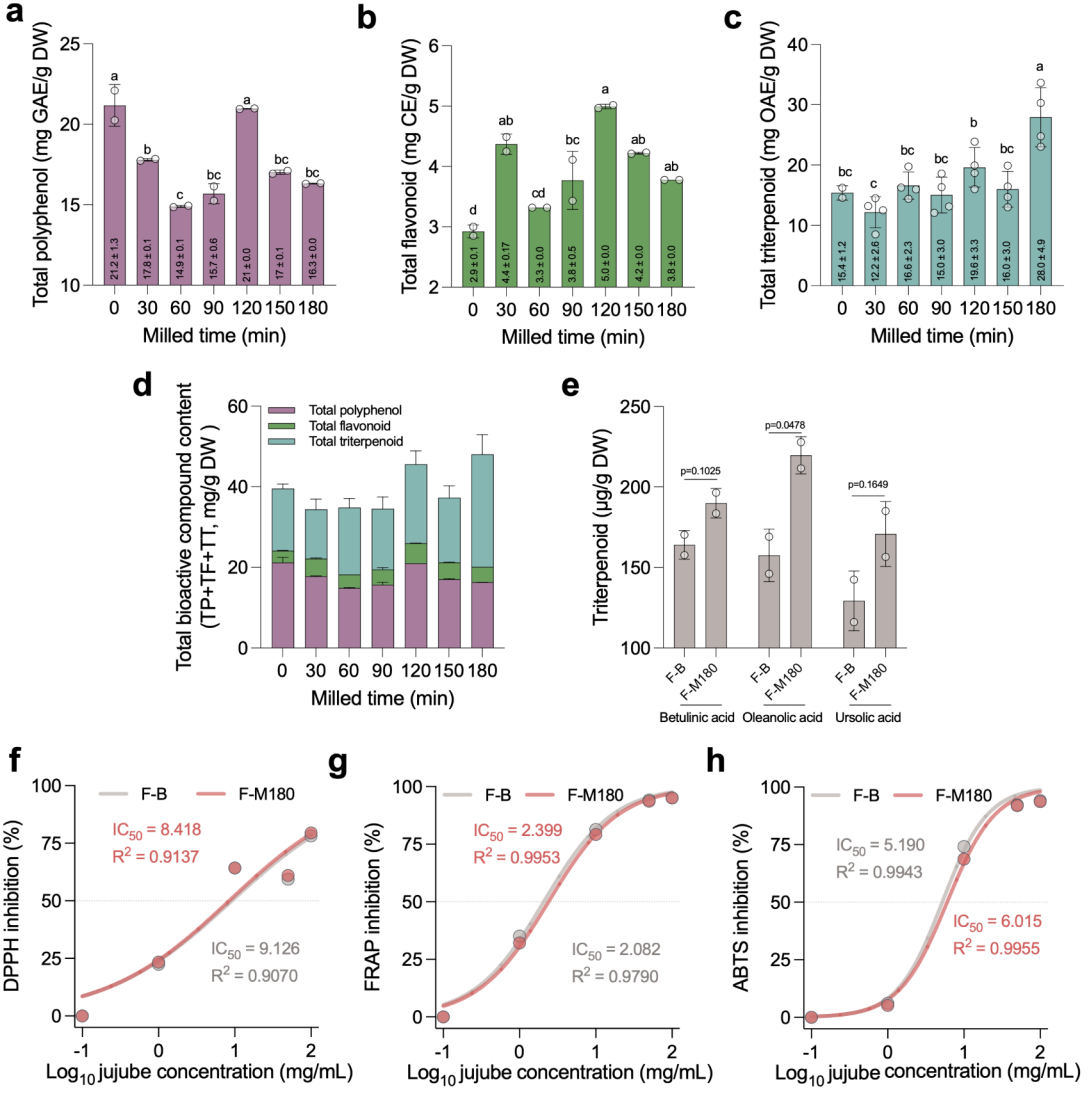
Effect of media milling on total polyphenol, flavonoid, triterpenoid content, and antioxidant activity of fresh jujube juice nanoparticles. (a) the total polyphenol content of jujube juice across different milling times (0–180 min), (b) total flavonoid content, (c) total triterpenoid content, (d) total bioactive compound content, including polyphenols, flavonoids, and triterpenoids, (e) individual triterpenoid components (betulinic acid, oleanolic acid, and ursolic acid) quantified in blended (F–B) and 180 min media-milled jujube juice (F-M180). (f) DPPH inhibition as a measure of antioxidant activity for F–B and F-M180, with IC50 values. (g) FRAP inhibition for F–B and F-M180. (h) ABTS inhibition for F–B and F-M180. Statistical analyses included one-way ANOVA with Tukey’s multiple comparison tests for panels (a), (b), and (c), and two-tailed Student’s *t* tests for (e). Dose–response curves (panels f–h) were analyzed using Log(inhibitor) vs normalized response – Variable slope models. Abbreviations: F–B, blended fresh jujube; F-M180, media-milled fresh jujube for 180 min. Different letters indicate significant differences (*p* < 0.05) among groups. Data are presented as mean ± SD.

Media milling significantly enhanced the bioactive compound content and antioxidant properties of fresh jujube juice (Figure 3). Total polyphenol, flavonoid, and triterpenoid contents increased with milling time, peaking at 120 min for polyphenols and flavonoids, and at 180 min for triterpenoids (Figure 3a–c). This enhancement can be attributed to the release of bound bioactive compounds through mechanical disruption of the cell walls. Combined bioactive compound content (TPC + TFC + TTC) was highest in media-milled samples, emphasizing the cumulative benefits of the milling process (Figure 3d). Quantification of individual triterpenoid components revealed significantly higher oleanolic acid content in media-milled juice (F-M180) compared to blended juice (F–B), underscoring the superior efficiency of media milling in releasing specific bioactives (Figure 3e). Antioxidant activity, evaluated through DPPH, FRAP, and ABTS assays, revealed free radical scavenging and reducing power in both F–B and F-M180, with F-M180 exhibiting lower IC50 values in DPPH inhibition compared to F–B (Figure 3f–h). These findings establish that media milling not only improves bioactive content but also translates to functional benefits in terms of antioxidant capacity.

### 3.4 Fermentation Dynamics of Media-Milled Jujube Juice

**Figure 4:**
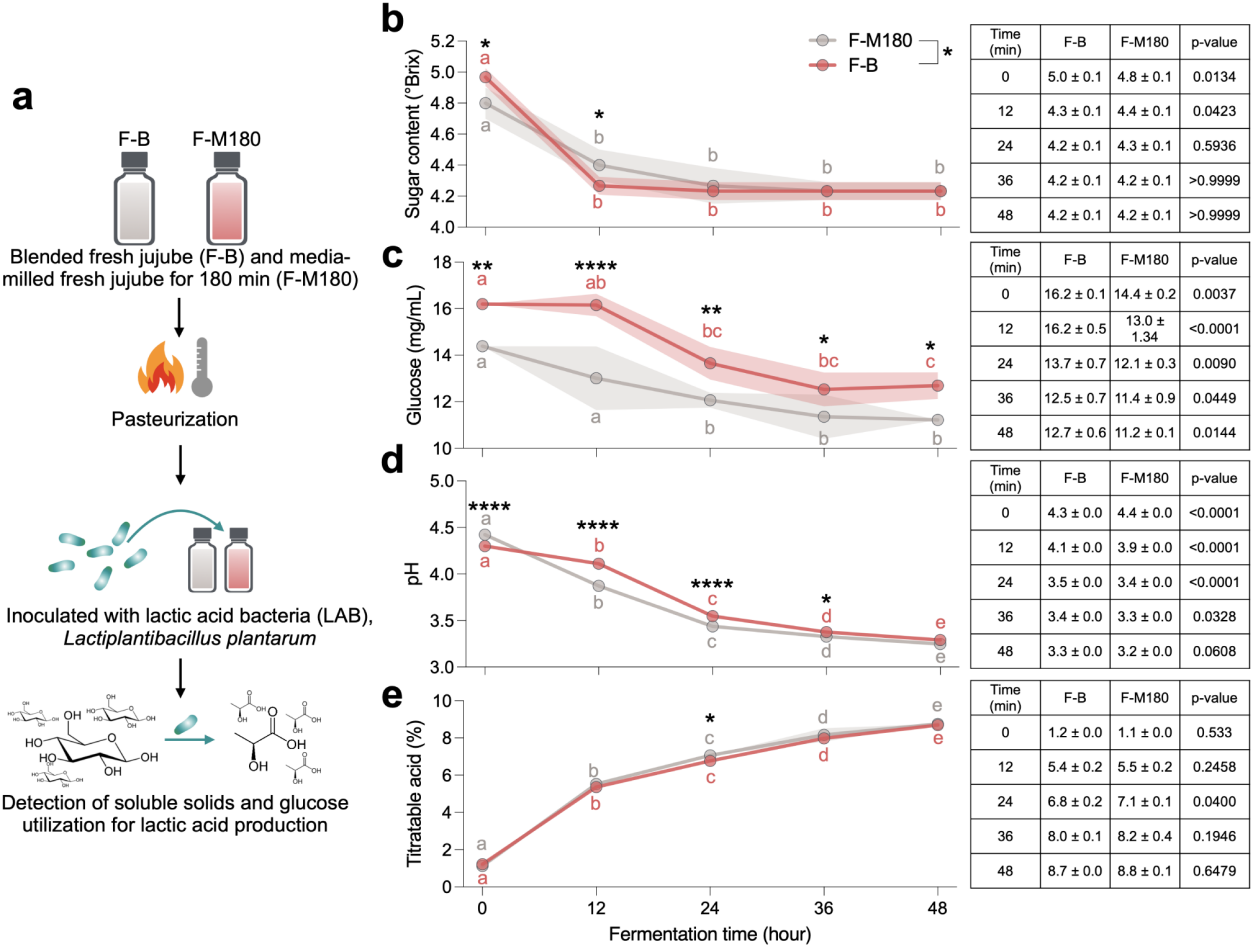
Utilization of blended fresh jujube (F–B) and media-milled fresh jujube for 180 min (F-M180) as substrates by *Lactiplantibacillus plantarum* for lactic acid production. (a) Schematic of the fermentation process: F–B and F-M180 were pasteurized and inoculated with *L. plantarum*. Changes in sugar content and glucose utilization were monitored to assess lactic acid production, (b) sugar content, (c) glucose concentration, (d) pH changes, (e) titratable acidity. Fermentation was carried out over 48 h. Statistical analyses were performed, with significant differences indicated by different letters (within-group comparisons at different time points) or asterisks (F–B vs F-M180; *p < 0.05, **p < 0.01, ****p < 0.0001). The results show efficient substrate utilization and acid production, with F–B and F-M180 showing distinct fermentation dynamics over time. Data are presented as mean ± SD.

The fermentation potential of media-milled jujube juice was assessed using *Lactiplantibacillus plantarum* (Figure 4a). Initially, the sugar content (°Brix) and glucose concentration in F-M180 were higher than in F–B. During fermentation, both sugar content and glucose concentration decreased significantly, with F-M180 demonstrating more efficient substrate utilization compared to F–B after 12 h of fermentation (Figure 4b,c). The faster depletion of sugars and glucose in F-M180 indicates that the nanoparticle structure of the juice enhances its availability as a substrate for microbial metabolism. However, the glucose content remained higher in F-M180 at all time points. Correspondingly, the pH decreased over time, reflecting increased lactic acid production, while titratable acidity increased, indicating a higher yield of organic acids in both the F–B and F-M180 groups (Figure 4d,e). These results highlight the suitability of both groups as substrates for lactic acid fermentation, potentially enhancing the functional properties of the resulting fermented products.

### 3.5 Anti-Inflammatory Activity in Macrophages

**Figure 5:**
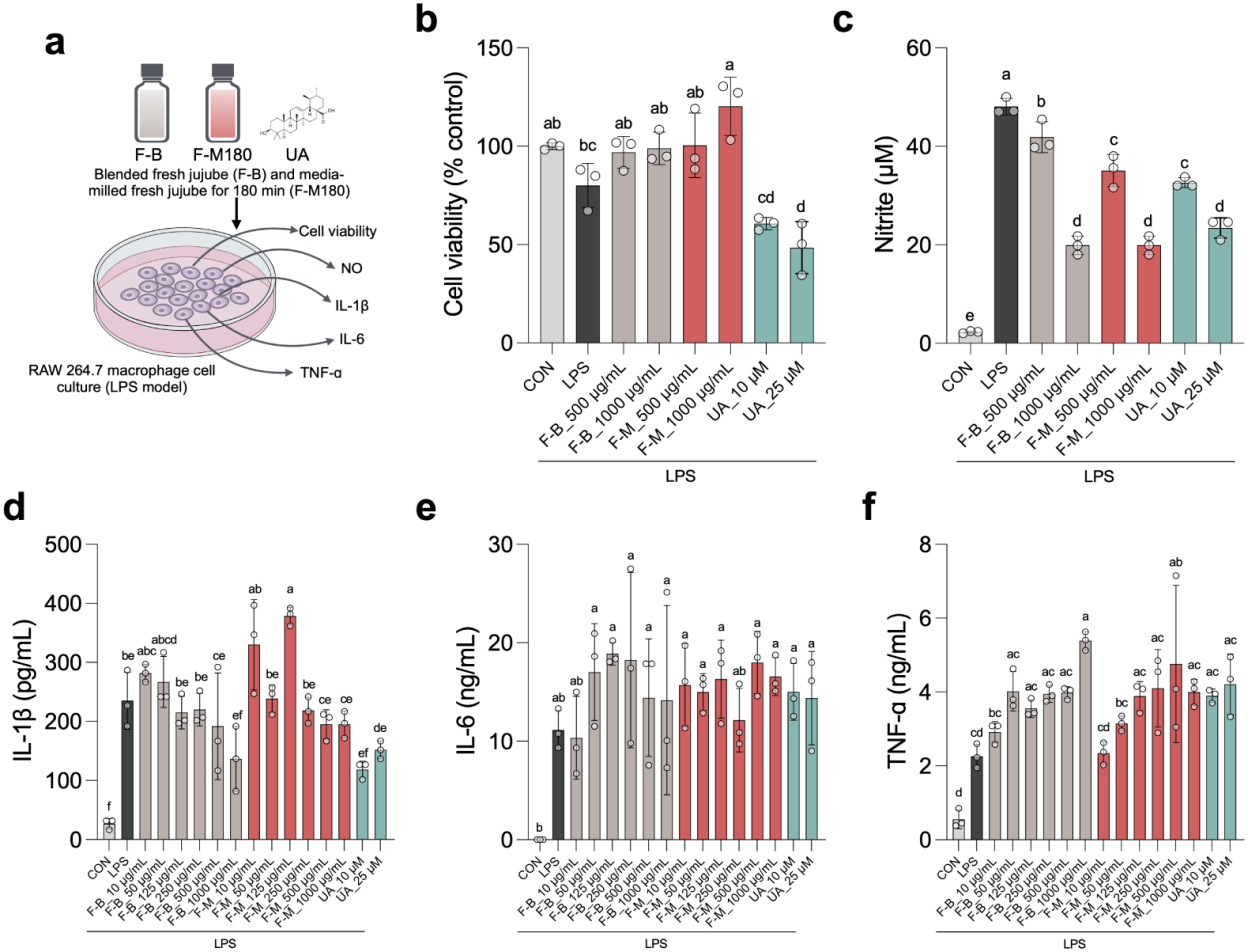
Effect of blended fresh jujube (F–B) and media-milled fresh jujube for 180 min (F-M) on RAW 264.7 macrophage cells stimulated by lipopolysaccharide (LPS). (a) Experimental design illustrating the treatments and key measurements, including cell viability, NO production, and cytokine levels (IL-1β, IL-6, TNF-α), (b) Cell viability, (c) nitrite (NO) production, (d) IL-1β secretion, (e) IL-6 secretion, and (f) TNF-α secretion. RAW 264.7 cells were treated with F–B, F-M, and ursolic acid for 24 h. Statistical analyses were performed using one-way ANOVA followed by Tukey’s multiple comparisons test. Different letters indicate significant differences (*p* < 0.05) among groups. Data are presented as mean ± SD.

The anti-inflammatory effects of media-milled jujube juice were evaluated in RAW 264.7 macrophages stimulated with lipopolysaccharide (LPS) (Figure 5a). F-M180 significantly preserved cell viability, indicating its nontoxic nature, and reduced nitric oxide (NO) production compared to F–B (Figure 5b,c). Pro-inflammatory cytokine level, IL-1β was significantly reduced in F–B and F-M180-treated cells, with effects comparable to those of ursolic acid, a well-known anti-inflammatory compound (Figure 5d,e). However, TNF-α and IL-6 levels showed no reduction in either group, and higher dosages may potentially increase TNF-α levels. These findings suggest that both F–B and F-M180 effectively modulate the inflammatory response, particularly by reducing IL-1β level, likely due to their higher bioactive content and antioxidant properties.

### 3.6 Protective Effects in DSS-Induced Colitis Mouse Model

**Figure 6:**
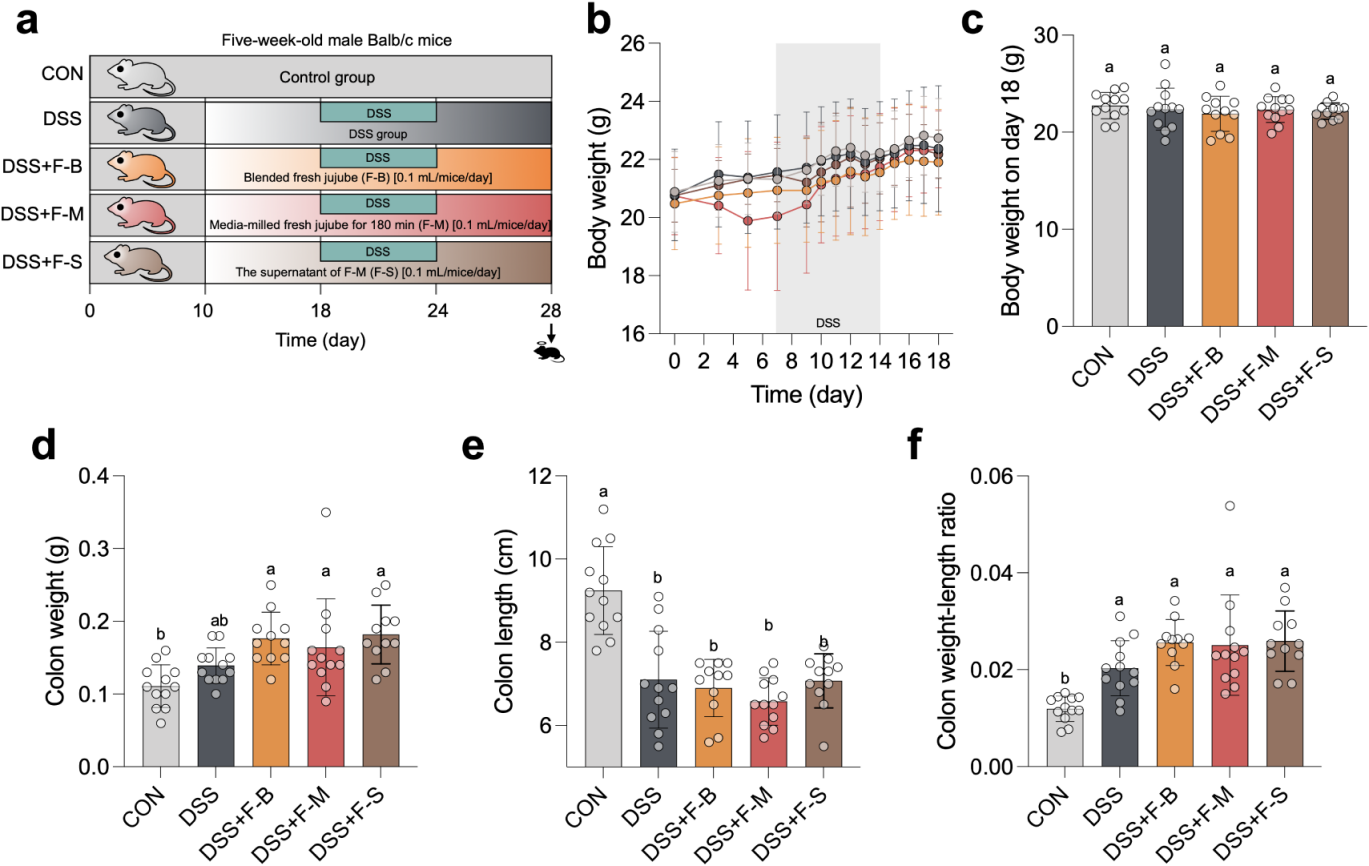
The impact of blended fresh jujube (F–B), media-milled fresh jujube for 180 min (F-M), and the supernatant of F-M (F–S) on body weight changes and phenotype in a DSS-induced colitis mouse model. (a) Schematic diagram of the experimental design, (b) body weight changes during the experimental period, (c) body weight on day 18, (d) colon weight. (e) colon length, and (f) colon weight normalized to body weight. Five-week-old male BALB/c mice were acclimatized for 10 days before receiving oral gavage with F–B, F–M, or F–S (0.1 mL/mouse/day) for 18 days. From day 18 to day 24, 2% DSS (dextran sulfate sodium) was added to the drinking water to induce colitis, followed by regular water until the mice were sacrificed on day 28. Statistical analyses were conducted using one-way ANOVA followed by Tukey’s multiple comparisons tests. Groups sharing the same letter are not significantly different (*p* > 0.05). Data are presented as mean ± SD.

The protective effects of media-milled jujube juice were evaluated in a DSS-induced colitis mouse model (Figure 6a). Five-week-old male BALB/c mice were acclimated for 10 days before receiving daily oral gavage with F–B, F–M, or F–S (0.1 mL/mouse/day) for 18 days. From day 18 to day 24, 2% DSS (dextran sulfate sodium) was administered in the drinking water to induce colitis, followed by regular water until the mice were sacrificed on day 28. The experiment included three groups to assess the impact of blended fresh jujube (F–B), media-milled fresh jujube for 180 min (F–M), and the supernatant of F–M (F–S). The inclusion of the supernatant group aimed to determine whether the functional compounds were present in the nanoparticle fraction or the supernatant. Body weight loss during DSS treatment, a hallmark of colitis severity, showed no significant differences among the groups (Figure 6b,c). Similarly, markers of inflammation severity, including colon weight, colon length, and the colon weight-to-length ratio, were not significantly different among the groups compared to DSS-only mice (Figure 6d–f). Although the treatments did not show protective effects, they also did not exacerbate colitis symptoms, suggesting the safety of blended and media-milled jujube juice at the administered concentrations. The lack of efficacy may be attributed to the low concentration of active compounds provided to the mice.

## 4 Discussion

This study investigated the impact of media milling on the physicochemical properties, bioactive compound content, and functional applications of fresh jujube juice. The results demonstrate that media milling significantly enhances nanoparticle formation, bioactive compound release, antioxidant capacity, and anti-inflammatory potential. These findings underscore the potential of media-milled jujube juice as a functional food ingredient with promising applications in health promotion.

The optimization of solid content and milling duration was critical for achieving the desired nanoparticle characteristics. A solid content of 5% and a milling time of 180 min yielded the smallest particle size with uniform dispersion, as evidenced by particle size analysis and microscopic observations. The volume-weighted average diameter decreased from 229.0 ± 1.0 μm to 25.0 ± 0.2 μm, while the number-weighted average diameter was reduced from 7.2 ± 0.0 μm to 0.1 ± 0.0 μm. This finding aligns with previous studies demonstrating that prolonged milling can effectively disrupt cellular structures, enhancing the release of intracellular components and improving suspension stability.^41^ The significant reduction in particle size and improved uniformity observed in this study provide a foundation for enhancing the functionality of jujube juice through nanoparticle technology.

Media milling was shown to improve the physicochemical properties of jujube juice, including stability, color, and viscosity. The increase in zeta potential and decrease in aggregation percentage highlight the enhanced stability of media-milled juice. These changes are likely due to the exposure of surface-active compounds during milling, which stabilize the nanoparticles and prevent sedimentation. Furthermore, the observed changes in color space variables (ΔE, L*, a*, b*) suggest that structural alterations in the juice components contribute to improved visual appeal. The increase in viscosity with prolonged milling can be attributed to the breakdown of insoluble components, which enhances the suspension’s consistency. These physicochemical improvements not only make media-milled jujube juice more appealing to consumers but also enhance its functional properties in downstream applications.

One of the most significant findings of this study is the enhanced release of bioactive compounds, including polyphenols, flavonoids, and triterpenoids, through media milling. The mechanical disruption of cell walls likely facilitates the liberation of these compounds, resulting in higher total bioactive content. This is consistent with previous reports suggesting that mechanical processes such as milling and homogenization can improve the bioavailability of bioactive compounds. The improved antioxidant activity observed in F-M180 samples further supports this hypothesis, as higher concentrations of polyphenols and flavonoids (*p* < 0.05) are known to enhance free radical scavenging and reducing power.^42^ These findings position media-milled jujube juice as a potent source of antioxidants, with potential applications in preventing oxidative stress-related diseases.

The enhanced fermentation dynamics observed in media-milled jujube juice highlight its potential as an optimal substrate for lactic acid bacteria (LAB). The rapid reduction in sugar content and glucose concentration during fermentation indicates that media milling improves the bioavailability of fermentable sugars.^16^ Furthermore, the increased titratable acidity and faster pH reduction in the samples reflect higher lactic acid production, which is essential for creating probiotic-rich fermented beverages. Previous studies demonstrating that pretreatment methods, such as milling, can enhance the fermentability of fruit substrates by LAB.^16,43^ The demonstrated suitability of media-milled juice for fermentation broadens its functional applications, particularly in the development of health-promoting fermented products. However, it is regrettable that this study found comparable fermentation performance between the F–B and F-M180 groups. This study focused on *Lactiplantibacillus plantarum* as a representative strain due to its consistent performance; however, future research should investigate multiple LAB strains to gain a broader understanding of fermentation dynamics and potential synergistic effects.

The anti-inflammatory effects of media-milled jujube juice observed in RAW 264.7 macrophages further underscore its functional potential. Both F–B and F-M180 significantly reduced NO production (*p* < 0.05) and pro-inflammatory cytokine levels IL-1β (*p* < 0.05), demonstrating their capacity to modulate inflammatory pathways. These effects are likely attributed to the higher concentrations of triterpenoids, polyphenols, and flavonoids in the media-milled juice. Our study observed a selective reduction in IL-1β levels, with no significant effect on TNF-α or IL-6. This selective reduction may be attributed to differences in the underlying signaling pathways, as IL-1β is primarily regulated by the NLRP3 inflammasome, whereas TNF-α and IL-6 are predominantly controlled by the NF-κB signaling cascade. Certain bioactive compounds in jujube juice, such as polyphenols and triterpenoids, may preferentially inhibit IL-1β production through targeted modulation of the inflammasome pathway. Further studies are warranted to confirm these molecular mechanisms.

Ursolic acid, a key triterpenoid, has been previously reported to exhibit anti-inflammatory properties by inhibiting NF-κB signaling and suppressing cytokine production.^44,45^ The comparable effects of F-M180 and ursolic acid observed in this study suggest that media-milled jujube juice could serve as a natural alternative for managing inflammation.

The DSS-induced colitis model provided in vivo evidence of the protective effects of media-milled jujube juice. F-M180 treatment mitigated body weight loss, improved colon length, and reduced inflammation-associated changes in colon weight. These findings are consistent with studies showing that bioactive compounds, such as polyphenols and triterpenoids, can modulate gut inflammation by enhancing barrier integrity and suppressing inflammatory cytokine production. The improved outcomes in F-M180-treated mice highlight the potential of media-milled jujube juice as a dietary intervention for managing inflammatory bowel disease (IBD) and related disorders. Importantly, the superior effects of F-M180 compared to blended juice emphasize the advantages of media milling in enhancing the therapeutic potential of functional foods.

The DSS-induced colitis model was used to evaluate the potential protective effects of media-milled jujube juice. However, F-M180 treatment did not mitigate body weight loss, improve colon length, or reduce inflammation-associated changes in colon weight and weight-to-length ratio compared to other treatment groups or DSS-only controls. These findings suggest that the administered concentrations of bioactive compounds, such as polyphenols and triterpenoids, were insufficient to exert a measurable impact on gut inflammation under the conditions tested. In addition, the absence of significant protective effects in the colitis model may be attributed to limitations in the dosing strategy and study design. This study employed a relatively low dose (0.1 mL/mouse/day) for 18 days, which may have been insufficient to achieve therapeutic outcomes. Increasing the dosage and extending the treatment duration in future studies could help clarify the potential benefits. Another possible limitation is the severity of the DSS-induced colitis model, which may have exceeded the protective capacity of the bioactive compounds in jujube juice. Future research should consider using a milder colitis model or combining jujube juice with other interventions to better evaluate its therapeutic potential.

While media milling has been shown in other studies to enhance the bioavailability of functional food components,^16,46^ the results of this study indicate that further optimization of dosing or formulation may be necessary to realize its therapeutic potential for managing inflammatory bowel disease (IBD) and related disorders. Notably, none of the treatments exacerbated colitis symptoms, confirming the safety of both blended and media-milled jujube juice at the tested concentrations. This study has limitations, such as the absence of histological analysis and molecular investigations at the genetic and protein levels, which could offer deeper insights into the anti-inflammatory mechanisms. Future research should focus on cytokine profiling and advanced molecular techniques to further elucidate these pathways. In addition, future studies should focus on analyzing gut microbiota composition to better understand the potential prebiotic effects of media-milled jujube juice and its impact on gut health.

The findings of this study have significant implications for the food and nutraceutical industries. Media-milled jujube juice represents a promising functional ingredient with applications in antioxidant-rich beverages, fermented products, and anti-inflammatory dietary supplements. The enhanced bioavailability and functionality achieved through media milling align with the growing demand for minimally processed, health-promoting foods. Furthermore, the demonstrated protective effects in a colitis model suggest potential applications in personalized nutrition and dietary management of chronic diseases. Future studies should explore the long-term effects of media-milled jujube juice in clinical settings and evaluate its synergistic potential with other functional ingredients.

## 5 Conclusion

Media milling has shown considerable promise as a technique to enhance the physicochemical properties, bioactive compound content, and functional capabilities of fresh jujube juice. This process significantly improved antioxidant activity, anti-inflammatory effects, and fermentation dynamics, emphasizing its potential for developing probiotic-rich fermented beverages and functional food products. Although the DSS-induced colitis model did not exhibit protective effects at the tested concentrations, the treatments did not worsen symptoms, underscoring the safety of both blended and media-milled jujube juice. These findings suggest that optimizing bioactive compound concentrations could further unlock the therapeutic potential of media-milled jujube juice for health promotion and disease prevention. This research provides a solid foundation for leveraging media milling to create innovative, health-focused jujube-based products.
